# The expression of awe: evolutionary origins and cultural elaborations of a basic emotion

**DOI:** 10.1098/rstb.2025.0148

**Published:** 2026-09-24

**Authors:** Dacher Keltner, Maria Monroy

**Affiliations:** ^1^ University of California, Berkeley, CA, USA; ^2^ Department of Psychology, Yale University, New Haven, CT, USA

**Keywords:** awe, expression, evolution, culture

## Abstract

Awe is the emotion people experience when encountering stimuli that are vast and beyond their current knowledge structures. In this article, we take a basic emotion approach to the study of the expression of awe, reviewing studies of awe-related movements of facial muscles, patterns of vocalization and bodily and postural movements. We first present evidence suggesting that awe indeed is a distinct emotional state. Guided by advances in the study of multi-modal emotional expression, we then review studies of how people express awe in a pattern of mouth and gaze activity, specific vocal bursts and head orientation upward, unfolding in coordinated patterns of expressive behaviour that have some degree of universality. Building upon recent theorizing in cultural evolution, we detail how the expression of awe is elaborated into cultural forms, including folklore, kinds of music (choral music), dance and portrayals of the sublime in visual arts.

This article is part of the theme issue ‘Mechanisms, development, phylogeny and functions of emotional expressions’.

## 1. Introduction

Awe is a brief emotion that people feel in response to what is most typically vast and beyond one's knowledge about the world [1]. Awe is often experienced in some of humanity's most elevated processes—in encounters with music, visual art, collective movement, for example, and in dance, ritual, sports, spiritual practice and contemplating big ideas. Might awe be expressed in evolutionary significant patterns of behaviour, in facial muscle movements, for example, or the sounds of the voice? This question presents something of a puzzle.

In one sense, awe is mysterious, transcendent, ineffable and beyond rational frameworks of understanding. Its experience in spiritual practice, or music, or in the context of a personal epiphany, for example, would seem to suggest that the experience of this emotion may not be associated with bodily expressions shaped by our evolutionary history. Perhaps awe is more akin to a purely cognitive or epistemological state, like flow or contemplation, not known to have accompanying expressive behaviour.

Recent theorizing about awe as a basic emotion, though, suggests that awe evolved to serve important social functions centring upon integrating the individual within social collectives. This evolutionary perspective locates awe in the body and suggests its experience might be accompanied by evolved patterns of signalling behaviour. Here, we review studies of the expression of awe in facial muscle movements, vocal bursts, shifts in gaze activity, head movements and bodily orientation. The discoveries generated in this line of inquiry allow us to consider the evolutionary origins of awe, as well as how it is elaborated into cultural expressions such as music, dance and visual art.

### (a) Awe as a basic emotion: assumptions and empirical progress

Within basic emotion theory (BET) emotions are thought of as distinct and brief states involving experiential, cognitive, physiological and expressive processes that enable humans to respond to evolutionarily significant problems, from negotiating status hierarchies to avoiding peril and disease to taking care of vulnerable offspring [2]. These core assumptions of BET have been foundational to a first wave of emotion science, guiding the study of the forms and functions of six emotions, namely anger, disgust, fear, sadness, surprise and joy (e.g. [3,4]).

Within BET, emotion categories like anger, embarrassment or compassion are not defined by essences in their response profiles [2,5]. Instead, they should be thought of as families of related states, which share core response patterning and meanings but vary according to individual differences and culture. This family-based perspective has been fruitful in understanding variations of embarrassment [6], pride [7] and compassion [8].

These two assumptions guide our approach to awe in this review (e.g. [1,9]). Namely, we posit that awe is a brief state that serves the function of enabling the individual to fold into groups and social collectives [9]. In keeping with this thinking, awe enables specific patterns of thought, such as the privileging of a more collective or communal self and patterns of action, such as sharing and cooperation, and related patterns of physiology, all of which enable the individual to integrate into social collectives, so vital to survival, reproduction and the raising of offspring to the age of viability (e.g. [1,9]).

Importantly, in keeping with a family-based approach found in BET, ‘awe’ as a concept encompasses a family of related states, with distinct ‘flavours’ [2,10,11]. People report feeling awe, for example, in quite different domains: in encounters with human virtues like kindness, in listening to music, in their relation to the Divine, and when thinking about big ideas, for example.

Awe also varies considerably according to the level of perceived threat that imbues the experience [11]. Awe-inspiring encounters with nature, for example, or in relation to cultural conceptions of the Divine, can be more or less threatening. Important work has shown that experiences of awe accompanied by greater degrees of threat differ from other experiences of awe in terms of the phenomenological qualia of the experience, the peripheral physiological profile and the benefits for well-being [12]. In cultures where threat is a more prominent theme in the experience of awe, the experience manifests in a different profile [13]. As we move through the review, it is important to recall that awe has different sources, from music to nature, and is flavoured by appraisals of threat, all of which should account for variations in its expressive behaviour.

Guided by this BET framework, relevant empirical science has begun to make the case that awe is a basic emotion, distinct in its response profile when compared to conceptually related emotions such as love (adoration), beauty, amusement, interest and fear. Namely, relevant empirical science has documented that awe is distinct in its subjective qualities [14,15]; central nervous system activation [16], which involves reduced activation in the default mode network [17]; and thought patterns and action tendencies (see [9]). More modest evidence is pointing to a peripheral physiological profile involving elevated vagal tone shared with other prosocial emotions [9].

Our cursory review suggests that awe is distinct from closely related states in terms of its experience, cognition, action tendency and, to a more modest degree, physiology. We now turn to its expressive behaviour, long a domain where claims about the forms and functions of emotion come into sharp relief.

## 2. The expression of awe

Beginning with Darwin, BET has been central to the study of emotional expression [2,5,18,19]. As this theorizing has evolved, it has led to a set of propositions that have guided relevant studies, and that will frame the ensuing review of the expression of awe: (i) an episode or experience of an emotion is associated with brief patterns of expressive behaviour; (ii) those patterns of expressive behaviour are recognized by outside perceivers; (iii) there will be some degree of cross-cultural similarity in both expression and recognition of the expressive behaviour; and (iv) other mammals, in particular social ones, will demonstrate similar behaviour in functionally equivalent contexts.

A first arc in the study of emotion expression began with the well-known studies of Ekman and Friesen of a limited number of prototypical facial expressions [20]. Emotion recognition studies, now numbering in the hundreds, have ascertained that people can recognize prototypical muscle configurations of six emotions—anger, disgust, fear, happiness, sadness and surprise [20–25]. There is considerable controversy about the strength of such effects [21,26–28].

The study of the expression of awe is part of a broader effort to map the expressions of emotions other than the six emotions of early interest to the field (for review, see [29]). The study of the expression of awe also reflects the movement beyond the face and to a new focus on multiple modalities of expression. Within emotion science, studies have begun to document the emotion-related meaning of gaze movements like looking down or upwards [30–33], head orientation up, down and to the side [34], posture [35], touch [36], dynamic body movements of the torso [37–40], vocalization [41–44] and full-bodied dynamic expressions involving the entire body, from head to the feet [45]. Some of this work has been replicated in cross-cultural studies [22,41,42,46–50].

Recognizing these advances, we now characterize the expression of awe, bringing evidence critical to claims that it is a basic emotion. We draw upon top-down approaches, where researchers begin with an emotion of interest—such as awe—and seek to document its patterns of expressive behaviour, for example in production studies, and how perceivers interpret limited and prototypical expressions. In more data-driven approaches, participants offer interpretations of vast arrays of more naturalistic, spontaneous expressions, and new statistical techniques identify the expressions that fall within a category of emotion like awe [51,52].

### (a) Patterns of awe-related facial muscle movements

Darwin [18] first proposed an awe-related facial expression that consisted of raised eyebrows and an open mouth, though he used the term ‘astonishment’. About a century later, Frijda [53] proposed a very similar expression for ‘amazement’. More recent empirical work has confirmed these as core components of awe's expression in the face.

In one emotion-production study, college-aged participants from five cultures—China, India, South Korea, Japan and the United States—were asked to express themselves freely while imagining themselves in twenty-two different, emotionally evocative situations [49]. The situation for awe involved the participant imagining a sudden encounter with a large and powerful waterfall. The facial muscle movements, gaze and head movements were then coded with a modified version of Ekman & Friesen's Facial Action Coding System [54], and statistical techniques then ascertained the ‘International Core Sequences’ (the actions observed in at least 50% of participants across five cultures) for the expressions of the 22 emotions.

This analysis revealed that across the five cultures, participants showed the following facial actions when displaying awe: raised eyebrows, smile, open mouth, jaw drop, and head movement backwards (see also [51,55,56]). With respect to our discussion of the BET approach to expression, individuals across the five cultures overlapped in 63% of the elements of the awe expression, suggesting significant cross-cultural similarity of the awe expression in this paradigm. The awe expression differed from the expression of interest, a closely related, epistemologically oriented emotion, which was expressed in a head nod across the five cultures. Importantly, culture-specific variation accounted for 14% of any randomly selected individual's expression, also suggesting clear cultural influences and that the expression of awe involves a family of patterns of expression. Awe did have systematic cultural variations, or dialects: in India, for example, the awe expression also included a lip pucker.

Based in part on these findings, Cowen and colleagues trained an AI model of identifying emotion from the face and applied it to over 2 million videos of spontaneous behaviour in emotionally evocative contexts of participants from 144 cultures [57]. The pattern of awe-related facial expression and head movement was systematically observed in response to seeing fireworks in the sky, and across the 144 cultures, the covariation between the expression and specific context was 70%, suggesting strong universality in the meaning of the awe expression.

**Figure 1.**
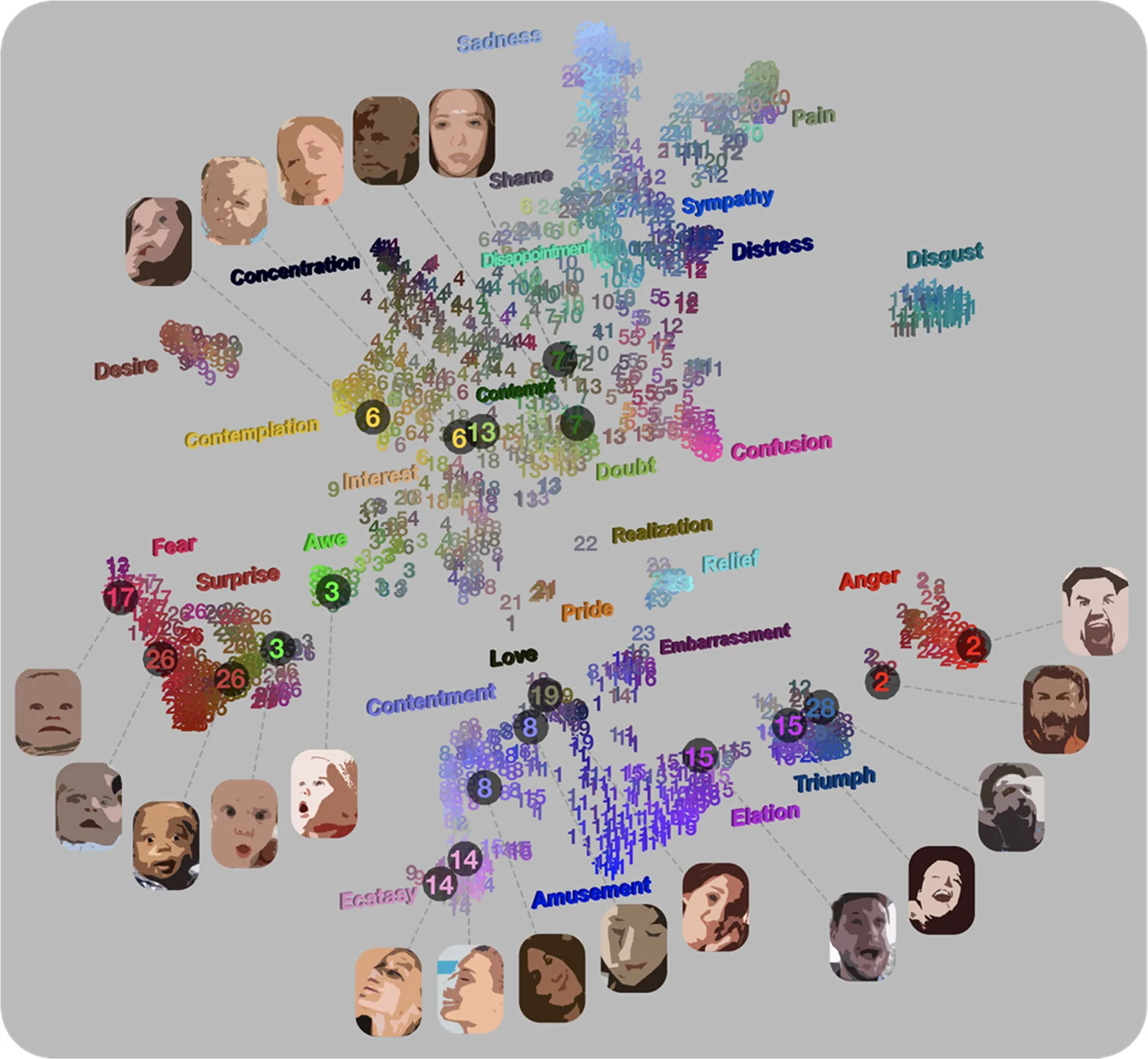
Map of the results.

Emotion recognition studies of the awe expression are limited, but suggestive. In one data-driven study, participants rated over 1500 naturalistic photos of facial–bodily expressions with over 40 terms related to emotion concepts (e.g. ‘embarrassment’) and appraisals [51]. In dimensionality reduction analyses, 28 emotion categories were required to explain participants’ judgements of facial–bodily expressions. A map of the results is presented in figure 1 (to explore this semantic space of facial expression, see https://s3-us-west-1.amazonaws.com/face28/map.html). As one can see, specific facial expressions were judged reliably by participants in this study as awe. Once again, perceivers differentiated between expressions of awe—involving a slight smile, open mouth, widened eyes and gaze upwards—as distinct from the facial expressions for related states like fear, surprise, ecstasy and interest.

This work on the awe-related facial expression is clearly preliminary. It will be important to document the patterns of facial muscle movement associated with the spontaneous experience of awe; the studies reviewed looked at facial muscle patterns produced when *imagining* being in an awe-related situation. It will be important for future research to examine the recognition of the facial expression of awe in emotion judgement studies involving diverse cultures.

### (b) Prosody and vocal bursts

The voice is a rich medium of emotional expression [19,46]. Studies of emotional expression in the voice can be characterized according to two methodological approaches.

In the first approach, people, often trained actors, are asked to express specific emotions like anger, fear, awe or joy in the tone, pitch, modulation and rhythm of speech—prosody—as they read a series of nonsense syllables or neutral passages of text, about the weather for example [42,46]. To ascertain the emotions expressed, these samples of prosody are then presented to listeners, who are asked to select the best match from an array of emotion-related terms for the emotion conveyed in the snippet of speech [58].

In one of the only studies of awe-related prosody, participants from India and the United States judged short samples of speech of participants from five countries, who were instructed to express different emotions through the tone of their voice. In this study, awe was one of the 12 emotions that participants from the two cultures reliably identified in short speech episodes, and they did so according to specific patterns of acoustic features [52]. Importantly, participants in India and the US converged considerably in their judgements of awe from prosodic variation in speech, as evident in the high cross-cultural correlations in the application of the label ‘awe’ to short episodes of prosody. Awe is communicated in prosody, with suggestive evidence that such expressions in the voice are similar across cultures.

The other prominent method in the study of the vocal expression of emotion focuses on vocal bursts—brief, non-word utterances that people emit vocally. Groans, snorts, spitting sounds and sighs are examples of vocal bursts, as are dozens of other kinds of non-word sounds. In this kind of research, people are presented with an evocative situation that produces an emotion (e.g. for awe, ‘you are seeing a large waterfall for the first time’) and asked to express that emotion with a brief sound but no words [43,44,59,60]. Vocal bursts of awe are often portrayed in sounds like ‘woa’ or ‘wow’ [41]. Vocal bursts captured in this way can then be played to a second group of participants—listeners—tasked with labelling each vocalization with one of many emotion terms, or to pick a situation from many that would be a fitting cause of the sound they just heard.

For example, Cordaro and colleagues presented vocal bursts of 16 emotions gathered from other participants to new participants in cultures from Western Europe, East Asia and Southeast Asia [41]. The participants in these cultures were asked to match emotionally rich, brief descriptions of situations (e.g. someone has insulted you; you hit your leg on a rock) to one of four vocal bursts of emotions of the same valence. Across emotions, participants reliably matched stories to the expected vocal burst 79% of the time. Participants proved to be able to reliably discern six positive emotions from vocal bursts—amusement, awe, contentment, desire, interest, relief and triumph—and six negative emotions—anger, contempt, disgust, embarrassment, fear, pain and sadness. The vocal burst of awe proved to be one of the most reliably identified emotion vocalizations across cultures.

### (c) Bodily movements

Expressive behaviour in the body adds to the richness of emotional expressions that one might observe in daily life. There is increased evidence suggesting that people can recognize different emotions in still body images [61] and dynamic body movements [35,37,38,40,62–64]. Relative to the face and voice, the body has been less well-studied though.

One study of full-bodied dynamic expressions of 34 emotional states, including those of awe, collected over 2700 natural (i.e. untrained) expressions from a first group of participants prompted, as in previous production studies, to express each emotion in their bodies in whatever way felt natural [45]. Over 29 000 recognition ratings of these naturalistic, full-bodied expressions were then gathered from a diverse group of new participants in the US. Importantly, this study involved significant numbers of female and male participants from three distinct cultural groups: Asian-Americans, Latin Americans and white European Americans.

Results from this study revealed that people were able to reliably recognize dynamic, full-bodied expressions of awe. Their accuracy rates in doing so were two and a half times greater than chance, and in this study, the recognition rates in identifying awe from full-body displays did not vary according to the ethnic background or gender of the individual expressing the emotion, nor the participant tasked with identifying the emotion, suggestive of cultural similarities in the expression and recognition of awe.

Subsequent analyses found that full bodied expressions of awe involve a slight head tilt upward, an expansive posture, open chest, upright torso, shoulders and arms relaxed (and less frequently, arms extended sideways or upwards or thrusted into the air), hands relaxed, open or on the chest (and small percentage of hands touching on face or head), and an overall stillness in the body as if to slow down and take in the experience. The body, this work suggests, is a modality of expression for a wide range of emotions, and in its movements can express awe to outside observers.

It is just as important to note the modalities in which awe does not seem to register in coherent and recognizable patterns of behaviour. Here we observe that while gratitude and compassion, two other-praising emotions, are readily communicated through brief tactile contact with a stranger's arm, this was not the case for awe [36].

### (d) Goosebumps

Emerging research on awe is beginning to identify the way in which this emotion affects the body, which may also be part of its expression, as the blush is, for example, for the emotion embarrassment. Early scholars linked awe to ‘the chills’ [65]. This description has since been refined to refer to a more specific experience of goosebumps and a tingling sensation or ‘goose tingles’, which has been documented across a variety of cultures during awe experiences [66]. Self-reported goose tingles occur more frequently during awe than other positive (e.g. interest) or negative (e.g. anger) states [66]. A daily experience method in which participants reported each time they felt goosebumps across a four-week period revealed that social experiences of awe, followed by aesthetic experiences, were the most common elicitors of reports of goosebumps (e.g. [67]), although actual laboratory inductions of awe were not found to elicit increased piloerection [68].

Piloerection in response to a more vast and often more powerful entity—so often the trigger of awe—may seem counter-intuitive given past claims that one function of piloerection in other mammals is to make the animal appear larger and more dominant [18]. However, one perspective on this apparent contradiction is that the initial reaction to objects or people that elicit awe may be one of fear or surprise, which is then replaced by a positive appraisal of one's safety and a curiosity about what one is encountering [67]. Therefore, goosebumps may represent an initial defensive reaction to something complex and not well understood. This subtle hypothesis has not yet been empirically tested.

Still other research suggests another possibility. Namely, goose tingles appear to co-vary, at least with respect to retrospective self-report, with positive, rewarding emotional experiences that predict closeness to another person, whereas cold shivers and the shudders co-vary with fear, disgust, threat and predicted social separation [66]. Therefore, this form of piloerection may simply represent a more positive experience theoretically distinct from dominance and threat. Whether goosebumps function to express the experience of awe to other observers has not been systematically studied.

## 3. Functions of the expression of awe: social signalling and cultural elaborations

Thus far, we have seen that across cultures, individuals express awe in a pattern of facial muscle movements—an open mouth, slight smile and widened eyes. In expressing awe, people recruit movements in their bodies—a head orientation upwards, a relaxing of the hands and torso and an opening of the hands. Perhaps the clearest expression of awe is in the voice—the *woa*s and *wow*s of awe. Very preliminary evidence suggests that there are cultural variations in the expression of awe as well.

We note clear limitations of this research. Most of the studies we have reviewed have examined the expression of awe in contexts of imagining being in nature or outside (viewing fireworks). The degree to which this pattern of expression replicates in studies of other variants or flavours of awe, for example in encounters with moral beauty or when listening to music, is not known. Nor is there evidence concerning how the threat-related flavouring of awe, known to influence the subjective feeling state of the experience as well as its central and peripheral nervous system profile, influences the expression of awe in the face, voice or body. One might imagine fear-related facial muscle movements or acoustic properties to shape the expression of threat-related awe. Finally, we note the scant evidence relating the spontaneous experience of awe to expressive behaviours.

From the forms of the expression of awe, we now move to a discussion of the functions of this expression. Within basic emotion theory, attention is given to the why of emotion—why do people express awe in this fashion? What signalling functions might such patterns of behaviour in the face, voice, body and gaze reflect?

One approach to this question is to offer accounts of why people engage in certain actions when feeling awe. On this point, scholars have posited that this expression may reflect or even facilitate enhanced information processing, most obviously through the opening of the eyes and the quieting or stilling of the body [18,69]. This would make sense given the fact that awe often is felt when encountering stimuli that are most typically mysterious, inexplicable and ineffable, thus requiring greater attention and concentration.

Another approach to the why of the awe expression is the study of its signalling functions, that is, what kind of information does the expression convey to others, and how does the transmission of that information alter the perceiver's behaviour [5,29]. Subjectively, when people feel awe, they report a sense of humility, a diminished focus on the self and the recognition of forces that are larger than the self [70,71]. This would suggest that observers should make clear inferences in encountering others’ expressions of awe, that the context they are in, for example, is mysterious, powerful or even involving forces larger than those encountered in social reality. This kind of thinking has been carefully applied to the study of the functions of the expressions of anger, embarrassment and pride, and should be fruitfully applied to the study of awe expressions.

Emotions also serve higher-level functions in how they are expressed culturally, for example in music, painting or dance [72–75]. This thinking is shaped by advances in cultural evolution theory, and the idea that cultural forms are repositories of knowledge that enable members of a culture to adapt more efficiently to the physical and social environment [72].

More specifically, the thesis is that cultural forms, such as stories, music, visual art, dance and ritual and ceremony, translate and deploy elements of emotions—patterns of expressive behaviour, sequences of social action or ways of seeing the world—in forms of culture such as painting, ritual, music or story. The expression of the emotions within such cultural forms allows individuals to share conceptual knowledge about emotions and, in doing so, arrive at a shared understanding of how emotions shape their shared social and moral life [72].

With respect to awe, scattered evidence finds that with language, people *represen*t experiences of awe in stories, legends, myths and metaphors, to share in an understanding with others what is vast and mysterious. This semantic structure of awe—that it follows encounters with what is powerful, vast and mysterious—is represented in stories about divine, spiritual and supernatural forces [76]. A systematic analysis of extraordinary experiences finds that these narratives focus on expressions of awe, ecstatic love and terror [77]. This line of thinking has also inspired recent studies of folklore—the fables, fairy tales and legends—which often represent encounters with what is vast and mysterious [78,79]. What has not been documented yet is how awe and closely related emotions, like interest or horror, structure narratives within such stories, and whether readers of such narratives readily identify the specific emotions they convey.

With visual techniques in paintings, carvings, masks and figurines, members of a culture represent emotionally rich interactions like childbirth, sexual relations, power dynamics (e.g. enslavement) and combat (e.g. [80]). Our work has found that one theme that pre-Colombian sculpture and carving conveys is encounters with the Divine [80], and the figures in such art look very much like facial and bodily expressions of awe. Similarly, in the visual arts, relevant data-driven research finds that people agree to a considerable degree in perceiving upwards of 20 emotions that are expressed in visual art, including awe, in paintings from different eras and across styles, suggesting that the expression of awe is a reliable theme in the visual arts [81].

Through *symbolization*, people dramatize expressions of awe in the voice, such as in singing, chanting and music, and in the bodily movements, such as in dance. One tradition of Hindu dance, detailed with remarkable precision in the Natyasastra, which is over 2000 years old, contains descriptions of how to perform and express more than 15 emotions in facial muscle movements, gaze activity and movements of the body and the hands. People from non-Hindu cultures, when presented with performances of different emotions via dance, have proven to be quite reliable in identifying the emotion expressed by the performer [82]. This includes portrayals of awe/wonder, which involves the following actions: eyebrows raised, eyes widened, an open mouth, a hand gesture to the mouth, a head tilt and a still, crouching posture. Westerners readily identified this as an expression of awe/wonder and differentiated it from portrayals of fear. Awe can be readily expressed in bodily movements as documented by Monroy and colleagues, but within aesthetic contexts like dance and theatre.

Perhaps earlier in human history, people began to express awe in singing, chanting and instrumental music. Alan Cowen and colleagues had participants from China and the US first provide minute-long musical selections that they personally felt expressed various emotions, including awe [83]. Chinese participants provided samples of traditional Chinese music. New participants from the two countries listened to samples of music and made judgements of the emotions they believed had been expressed in the music. These participants could reliably detect 13 emotions in the musical selections, including those provided by individuals from the other culture. Of note, US participants agreed with Chinese participants in their judgement of the emotions expressed in selections of traditional Chinese music. Of the 13 emotions that members of both cultures agreed upon in their perceptions of the feelings expressed by music, one was awe.

The musical selections that expressed awe, it is noteworthy to observe, shared acoustical features that resembled the *woa*s*, wow*s and *ahh*s people vocalize when feeling awe. This is in keeping with theorizing that holds that music expresses specific emotions by deploying sounds and acoustic features of emotion-related prosody and vocal bursts [42]. In a more speculative vein, it has been noted that vocalizations of awe are deployed in sacred chanting that one observes in cultural traditions in different parts of the world [84].

Finally, through *ritualization*, people elaborate upon simple emotion-related behaviours into collectively performed, repetitive acts with shared significance, often in contexts that establish and signal an individual's collective identity [85,86]. One recent line of studies found that awe-related bowing, arms thrust into the air and touching ‘the sacred’ resemble specific rituals that are elements of religious ceremony [86,87]. These expressions of awe, which can be considered the constitutive aspects of rituals, are often experienced in synchrony with others in a collective effervescence [88,89].

In this new line of theorizing, cultural practices like folklore, music and dance and ritual allow members of a culture to express the emotions that are vital to human social life. As we have shown here, cultural elaborations upon our evolved expressions of awe in facial muscle movements, vocalizations, gaze activity and head and body movements can be seen in artistic expressions, including story, visual art, music, dance and ritual, that are perceived as expressions of awe. Through such expression, people come to share in an understanding of the emotion, and in the case of awe, what is vast, mysterious and beyond human understanding, and that unites us into collectives.

## 4. Awe as a basic emotion?

The science of awe has expanded dramatically in the past 15 years, with a focus on its benefits for health and well-being [1,9] and its forms and functions. With precedent in the study of other emotions, in this article, we considered the evidence for the claim that awe is a basic emotion with distinct experience, thought, physiology and, our particular concern here, expressive behaviour.

Within BET, distinct emotions are claimed to have distinct patterns of expressive behaviour that are recognized by others and demonstrate some degree of cross-cultural similarity. The evidence we have reviewed does indeed find a clear pattern to the expression of awe, involving: an open mouth and movements of the eyes upwards; specific prosodic features and vocal bursts; and movements of the head, chest, arms and stillness of the body. Large-scale studies of the face and voice, and more traditional emotion recognition studies have unearthed some evidence for the universality of this expression of awe. Awe can also be expressed in story, painting, music, sculpture and dance. We note that few, if any, studies have looked at patterns of expressive behaviour associated with spontaneous experiences of the emotion.

The search for distinct emotions guided by BET has been a central enterprise in affective science and opened the field up to the study of moral and aesthetic emotions. The work we have reviewed here provides preliminary evidence for the thesis that awe is a distinct and basic emotion, a thesis that awaits further study of how this emotion unfolds.

## Data Availability

This article has no additional data.

## Theme

One contribution of 19 to a theme issue ‘Mechanisms, development, phylogeny and functions of emotional expressions’.
